# Pathogenesis and Immune Response Caused by Vector-Borne and Other Viral Infections in a *Tupaia* Model

**DOI:** 10.3390/microorganisms7120686

**Published:** 2019-12-12

**Authors:** Mohammad Enamul Hoque Kayesh, Md Abul Hashem, Bouchra Kitab, Kyoko Tsukiyama-Kohara

**Affiliations:** 1 Transboundary Animal Diseases Centre, Joint Faculty of Veterinary Medicine, Kagoshima University, 1-21-24, Korimoto, Kagoshima-city, Kagoshima 890-0065, Japan; mehkayesh@yahoo.com (M.E.H.K.); mdhashem29@yahoo.com (M.A.H.); bouchra.kitab17@gmail.com (B.K.); 2 Department of Microbiology and Public Health, Faculty of Animal Science and Veterinary Medicine, Patuakhali Science and Technology University, Barishal 8210, Bangladesh

**Keywords:** immune response, *Tupaia*, viral infections

## Abstract

The *Tupaia* or tree shrew (*Tupaia belangeri*), a small mammal of the Tupaiidae family, is an increasingly used and promising infection model for virological and immunological research. Recently, sequencing of the *Tupaia* whole genome revealed that it is more homologous to the genome of humans than of rodents. Viral infections are a global threat to human health, and a complex series of events are involved in the interactions between a virus and the host immune system, which play important roles in the activation of an immune response and the outcome of an infection. Majority of immune response data in viral infections are obtained from studies using animal models that enhance the understanding of host-virus interactions; a proper understanding of these interactions is very important for the development of effective antivirals and prophylactics. Therefore, animal models that are permissive to infection and that recapitulate human disease pathogenesis and immune responses to viral infections are essential. Several studies have shown the permissiveness of *Tupaia* to a number of important human viral infections in vitro and in vivo without prior adaptation of the viruses; the immune responses and clinical manifestations were comparable to those observed in human infections. Thus, the *Tupaia* is being utilized and developed as a promising immunocompetent small animal model for viral infection studies. In this review, we focused on the immune responses, mostly innate, during viral infection and pathogenesis in the *Tupaia* model; we evaluated the interaction between the virus and the components of host resistance, the usefulness of this model for immunopathogenesis studies, and the vaccines and antivirals available.

## 1. Introduction

The investigation of the immune response to viruses has provided fundamental insight into the functioning of the host’s immune system. Interactions between viruses and the host immune system involve a complex series of events that may lead to the activation of the immune response and the clearance of the infecting agent. The use of small animal models is an important system for understanding these events that occur when viruses interact with their host, which is important in the development of therapeutic approaches. The innate immune response safeguards the host against viral infections as the first line of defense, and early and non-specific detection of pathogens are generally mediated by the recognition of pathogen-associated molecular patterns (PAMPs) by different pattern recognition receptors (PRRs), such as Toll-like receptors (TLRs), retinoic acid-inducible gene I (RIG)-like receptors (RLRs), nucleotide-binding oligomerization domain-containing protein (NOD)-like receptors, C-type Lectin, and DNA-sensing receptors [1,2]. Toll-like receptors (TLRs), an important component of the host innate immune system, play a significant role in sensing invaders and modulating innate and adaptive immune responses, limiting the spread of infections [3,4]. Recently, cyclic GMP-AMP (cGAMP) synthase (cGAS) has been recognized as a cytosolic DNA sensor and an important element in the induction of innate immune responses. [5,6]

Animal models for human viral infections are important tools for characterizing viral pathogenesis and host-virus interactions. A significant number of animal models have been used in viral pathogenesis and immune response studies, but each model has its advantages and disadvantages. Therefore, it is important to investigate alternative suitable animal models for host-virus interaction and pathogenesis studies. The Northern tree shrew (*Tupaia belangeri*) in the Tupaiidae family is a small mammal, similar in appearance to squirrels, with a bodyweight ranging between 100 and 150 g [7]. Tree shrews are native to southwest Asia [8]. The *Tupaia* whole genome has been sequenced [9,10] and phylogenetic analysis based on whole genome sequences revealed that the genome of *Tupaia* is more similar to that of humans than they are to mice [7]. The high degree of genetic homology between several neuromodulator receptor proteins in tree shrews and primates enabled the expanded use of *Tupaia* in preclinical research, and thus, *Tupaia* have been used in a variety of research fields [11,12]. In infectious disease research, the *Tupaia* has appeared as a promising candidate animal model and has shown susceptibility to several important human viral pathogens, including *Hepatitis B virus* (HBV) [13,14,15,16], *Hepatitis C virus* (HCV) [17,18,19], *Hepatitis E virus* [20], *Influenza virus* [21,22,23], *Dengue virus* [24], *Zika virus* [25,26], *Epstein–Barr virus* [27], herpes simplex virus [28], *Coxsackie virus* A16 [29], Newcastle disease virus, and *Sendai virus* [30]. Construction of a complete *Tupaia belangeri* transcriptome database by whole-genome and comprehensive RNA sequencing may facilitate a better understanding of viral pathogenesis and host immune responses [10]. Animal models of human viral illnesses are needed in order to generate safe and effective antivirals and vaccines. Therefore, in this study, we aimed to address the findings obtained from research on human viral infections using the *Tupaia* model, which might help to elucidate the underlying mechanisms of host-virus interactions and viral pathogenesis. This may provide an opportunity to evaluate virus-host interactions, host defense mechanisms, immunopathogenesis, and the efficacy of vaccines and antivirals in this animal model.

## 2. *Dengue Virus* (DENV)

**Figure 1:**
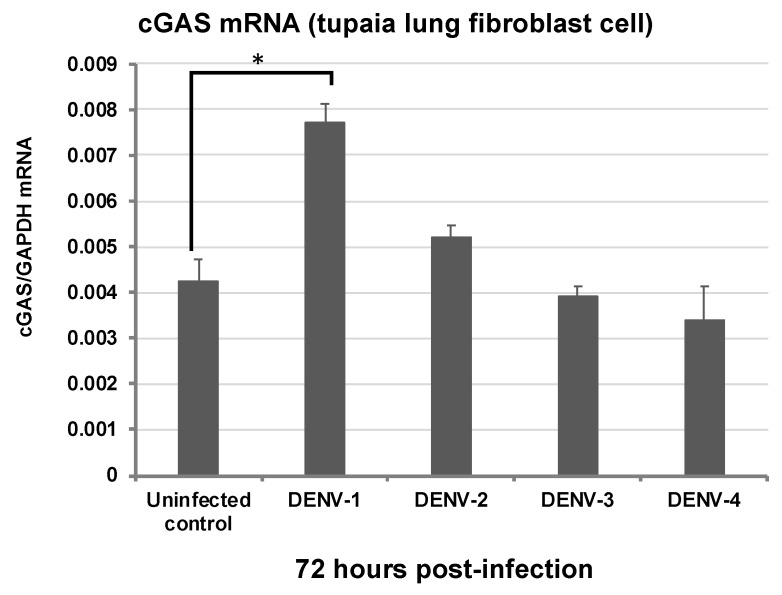
Expression of cGAS mRNA in *Tupaia* cells infected with DENV-1, DENV-2, DENV-3, and DENV-4 72 h post-infection (multiplicity of infection (MOI = 1)) measured by one-step qRT-PCR [16]. Gene expression levels were normalized against the expression level of GAPDH mRNA. Asterisk indicates significant differences calculated by the Student’s *t*-test (* *p* < 0.05). Where not indicated, differences were not significant (*p* > 0.05). Data are presented as mean ± SD (*n* = 3).

*Dengue* is the most prevalent and rapidly spreading mosquito-borne viral disease, rendering 3.9 billion people at risk of DENV infection worldwide, with around 390 million DENV infections occurring each year [31]. There are four different but closely related DENV serotypes: DENV-1, DENV-2, DENV-3, and DENV-4. Their infections result in a wide range of clinical manifestations ranging from self-limiting *Dengue* fever to life-threatening *Dengue* hemorrhagic fever and *Dengue* shock syndrome [32]. Currently, licensed vaccines for common use or antivirals are lacking, impeding the prevention and control of DENV infection. Progress towards understanding DENV pathogenesis and immune response is hampered by the lack of a suitable animal model, which can reflect *Dengue* diseases in humans [33]. Animal models, including mice, rhesus monkeys, chimpanzees, and marmosets, have been utilized in studying DENV infection but their ability to recapitulate the human disease remains challenging [33]. Therefore, searching for alternative suitable small animal models for DENV infection study is imperative. To this end, we have investigated the susceptibility of *Tupaia* cells to infection by wild type DENV serotypes 1–4 [24]. All the serotypes could establish infection in *Tupaia* cells and viral copy numbers increased linearly after the onset of infection, with DENV serotype 2 showing the highest replication efficiency in the cells [24]. As the role of toll-like receptors (TLRs) on innate immune recognition against DENV infection was not well characterized, we used *Tupaia* cells to characterize the role of TLRs in DENV infection; an increase of TLR8 messenger RNA (mRNA) levels was found in all DENV infections. Knockdown of TLR8 expression led to a significant increase in DENV-1 viral load, both in *Tupaia* cells and their corresponding culture supernatants, indicating a suppressive role of *Tupaia* TLR8 in DENV replication in vitro [24]. In addition, we also observed modulation of cytokines/chemokines in DENV-infected *Tupaia* cells, suggesting the involvement of these innate immune components in DENV infection, similar to that observed in human infections [34,35,36]. A recent study [37] demonstrated that DENV could activate a cGAS response and in an in vitro study of DENV infection we detected the expression pattern of cGAS in the sample from a previous study [24], as described [16]. We observed that DENV serotype 1, but not other serotypes, significantly upregulated cGAS mRNA expression 72 h post-infection in *Tupaia* cells (Figure 1). This may suggest different features of DENV serotype 1 compared to the other serotypes.

Notably, we also performed in vivo inoculation of DENV serotype 2 in the *Tupaia* and observed that DENV could replicate well. Induced anti-DENV antibodies were observed 2 weeks post-infection (our unpublished data). Therefore, the findings from our previous studies suggest that the *Tupaia* could be investigated as an immunocompetent animal model for DENV pathogenesis studies and the development and evaluation of antivirals and vaccines.

## 3. *Zika Virus*

*Zika virus* (ZIKV), an emerging arbovirus, is a positive-sense single-stranded RNA virus of the Flaviviridae family, along with other important human pathogens including *Dengue virus* (DENV), yellow fever virus (YFV), *West Nile virus* (WNV), Japanese encephalitis virus (JEV), and tick-borne encephalitis virus (TBEV) [38]. After being first reported in Uganda in 1947 [39], the spread of ZIKV did not draw much attention for over half a century. Surprisingly, ZIKV re-emerged in 2016 in Brazil and has spread quickly worldwide [40]. Due to the worldwide transmission and deleterious clinical outcomes of ZIKV infection, WHO recently declared ZIKV fever as a public health emergency [41] and the virus has since received global attention. ZIKV is transmitted by *Aedes* mosquitoes and causes symptoms similar to those caused by many other mosquito-borne flaviviruses including fever, rash, arthralgia, conjunctivitis, and muscle aches [38,42]. In addition, ZIKV can cause severe congenital diseases as a result of maternal infection during pregnancy [43]. Different animal models, including mice, guinea pigs, hamsters, and non-human primates, have been utilized for characterizing the biology and pathogenesis of ZIKV [44]. A better understanding of ZIKV biology and pathogenesis is necessary for the development of antivirals and vaccines for the control and prevention of ZIKV infection. Although the animal models previously utilized in ZIKV infection have advanced our understanding of the biology and pathogenesis of ZIKV infection, dermatological manifestations are rarely recapitulated in these animal models [25]. Recent studies have shown the susceptibility of *Tupaia* to ZIKV infection [25,26]. An in vitro study demonstrated tissue tropism of ZIKV infection in tree shrew primary cells from the kidney, lung, liver, skin, and aorta. These ZIKV-infected cells were able to support viral replication, with skin fibroblast and vascular endothelial cells showing high sensitivity to the infection [26]. Accordingly, an in vivo study demonstrated notable dermatological manifestations such as skin rashes in ZIKV-infected *Tupaia*, which are commonly observed in human patients [25]. The primary dermatological manifestations in human ZIKV infection are maculopapular rash and pruritis [42,45]. ZIKV infection in primary *Tupaia* cells induced an upregulation of cytokine mRNA levels over the infection time, including those of IL-6, IL-8, TNF-α, IFN-β, CXCL9, and MX1, where IL-6, IL-8, and TNF-α mRNA levels were significantly elevated 6 h post-infection [26]. The induction of an immune response in *Tupaia* following ZIKV infection has also been reported [25]. In addition, ZIKV infection could not be established in previously infected *Tupaia* re-infected with the same type of ZIKV, suggesting that ZIKV was inhibited by neutralization antibodies produced during the first infection [25]. Therefore, the *Tupaia* model could be a suitable animal model for the investigation of disease symptoms in ZIKV infection, which might help to elucidate ZIKV pathogenesis and lead to the development of novel vaccines and therapeutics. Notably, the *Tupaia* model has shown the potential to be an alternative animal model for viruses in the *Flaviviridae* family, more precisely, for species from the flavivirus genus, such as DENV and ZIKV. It is also important to investigate the susceptibility of *Tupaia* to other members of the flavivirus genus, like *West Nile virus*, Japanese encephalitis virus, etc., which may open a new window to characterize the pathogenesis and immune response of flavivirus infection in this immunocompetent and suitable small animal model for the elucidation of many unanswered issues.

## 4. *Hepatitis C Virus* (HCV)

**Figure 2:**
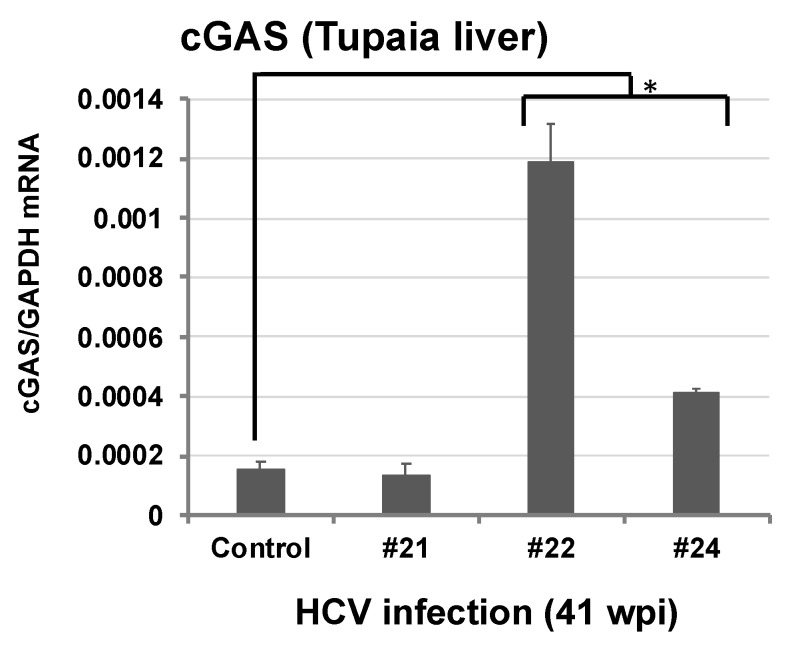
Intrahepatic *Tupaia* cGAS response to HCV infection 41 weeks post-infection. Changes in the expression levels of cGAS mRNAs in HCV1a- (#21), HCV1b- (#22), and HCV2a- (#24) infected *Tupaia* liver tissues measured by one-step qRT-PCR [16]. Gene expression levels were normalized against the expression level of GAPDH mRNA. Asterisk indicates significant differences calculated by the Student’s *t*-test (* *p* < 0.05). Where not indicated, differences were not significant (*p* > 0.05). Data are presented as mean ± SD (*n* = 3).

HCV is a major public health problem with approximately 130–170 million people affected worldwide. This virus is also a leading cause of chronic liver disease, cirrhosis, and hepatocellular carcinoma [46,47]. The lack of a suitable small animal model is a big obstacle in the field of HCV research. So far, chimpanzees have been used as a natural infection model for HCV. However, high costs and ethical concerns have restricted chimpanzee use in experimental infections. Although animal models like humanized chimeric mice [48] and genetically humanized mice [49] have been used as HCV infection models [48,50], these models have limitations, including high cost, immunocompromised animal status, donor-to-donor variability, and the inability to examine chronic infections. Therefore, alternative animal models are essential for a better understanding of the viral pathogenesis and host immune response for the development of vaccines; further, a vaccine against HCV infection is yet to be developed. Several studies have demonstrated the susceptibility of *Tupaia* to infections with different HCV strains, where the disease course of chronic HCV infection was comparable to that observed in humans. However, sustained/intermittent propagation of HCV with medium/low viral loads were observed in *Tupaia*, which might be associated with a more restricted replication of HCV in *Tupaia* compared to humans [17,18,19]. In chronic HCV infection, histopathological findings, including lymphocytic infiltration, disturbance of hepatic cords, and initiation of fibrosis in the liver of HCV-infected *Tupaia* were observed; these findings were similar to those observed in human patients [19]. Previous studies have reported the development of liver cirrhosis and hepatocellular carcinoma in *Tupaia* during HCV chronic infection [18]. In addition, Amako et al. showed that infection of naïve animals with viral RNA-positive *Tupaia* serum could establish re-infection, which was indicated by the development of acute hepatitis and viremia, suggesting that HCV infection in *Tupaia* could reproduce the pathogenesis typically associated with acute and chronic hepatitis [18]. In a recent study, we demonstrated the generation of anti-core and anti-NS3 antibodies in HCV infection in a *Tupaia* model, which suggests the potential of using *Tupaia* in vaccine experiments [19]. In the same study, we also characterized TLR mRNA expression, and similar to studies in humans, we found increased intrahepatic levels of TLRs 3, 7, and 8 [19]. Remarkably, HCV infection in *Tupaia* triggered ROS generation in sera and liver tissues and subsequently induced anti-3β-hydroxysterol-Δ24-reductase (DHCR24) antibody production [19], similar to observations in human infections [51,52,53,54,55]. A previous study demonstrated anti-DHCR24 auto-antibodies as a biomarker for *Hepatitis C* progression [56] and the findings of HCV chronic infection in *Tupaia* also supported the possibility of using anti-DHCR24 antibodies as a valuable marker to monitor HCV infection in vivo. It was reported that the 3′ untranslated region (UTR) of HCV can trigger a STING-dependent response in hepatocytes [57]. Using the liver samples from a previous study [19], we determined the intrahepatic expression pattern of cGAS during HCV chronic infection in *Tupaia* as described previously [16]. Significant upregulation of cGAS mRNA was observed in HCV1b- and HCV2a-infected *Tupaia* (#22 and #24, Figure 2) but not HCV1a (#21, Figure 2). This may highlight the difference in innate immune responses among the HCV genotypes.

Overall the findings of studies using the *Tupaia* model have advanced our understanding of the molecular pathogenesis of HCV infection and host immune responses. These findings may facilitate host immune response characterization in HCV infection, which may play a significant role in developing the *Tupaia* as an immunocompetent animal model for HCV infection and the development of antivirals.

## 5. *Hepatitis B Virus* (HBV)

HBV is a major causative agent of chronic hepatitis, liver cirrhosis, and/or hepatocellular carcinoma. HBV is an enveloped, circular, and partially double-stranded DNA virus [58]. This virus is a global public health threat with over 248 million people suffering from chronic HBV infection worldwide [59,60]. HBV displays a high specificity for its hosts, and besides humans, chimpanzees have been used as an infection model for HBV. However, due to economic and ethical reasons, the use of chimpanzees for experimental infection is difficult. The *Tupaia* is the only non-primate animal that shows susceptibility to human HBV infection and several studies have demonstrated the utility of the *Tupaia* as an immunocompetent animal model for HBV infection [13,15,16]. In addition, Ruan et al. demonstrated the similarities of pathological changes in HBV-infected *Tupaia* liver tissues with the disease progression in humans [61]. In a recent study, we found that the establishment of chronic HBV infection and an impaired IFN-β response could be caused by the HBV-A2 genotype in the *Tupaia* infection model [16]. On the other hand, during acute infection, impaired IFN-β response was only observed in HBV-A2_JP4-infected *Tupaia*, but not in HBV-A2_JP1-infected *Tupaia*, which is suggestive for the role of IFN-β suppression towards the establishment of chronicity in the *Tupaia* model of HBV infection [16]. In another study, Kouwaki et al. demonstrated that IFN-γ mRNA expression was significantly induced in *Tupaia* liver tissues during HBV-C infection at 1 or 3 days post-infection, but IFN-β was not [62]. This observation is indicative of a complicated relationship between HBV and innate immunity in vivo. Further, a higher replication efficiency of HBV-A2 than HBV-C was observed in *Tupaia*, which is suggestive of the genotype-specific replication nature of HBV in the *Tupaia* model [16]. Additionally, we investigated the expression levels of cGAS, which was found to be induced in all *Tupaia* at 28 days post-infection and partially induced at 31 weeks post-infection [16], suggesting that suppression of cGAS might have a role in the development of chronicity. Interactions between HBV and TLRs have been clearly demonstrated in a recent study [63]. In our study, we observed significant upregulation of intrahepatic TLR2, TLR3, TLR4, TLR8, and TLR9 mRNA expression in HBV-A2_JP1-infected *Tupaia*. However, the TLR response was found to be suppressed in chronic infection, indicating HBV and TLR interactions in the *Tupaia* infection model of HBV [16]. TLR9 mRNA expression was found to be suppressed in HBV chronic infection in the *Tupaia* model, which is consistent with a previous report showing reduced TLR9 expression in peripheral CD14+ monocytes of chronic hepatitis B patients [64]. This also may indicate that HBV might have different arms to resist the innate immune system during different periods of infection. Thus, future studies with animal models that support efficient HBV infection are necessary to further investigate the modification of TLRs by HBV infection. Establishment of an adaptive strain producing high viral loads in the *Tupaia* model may further contribute to the elucidation of pathogenesis and immune response for the development of antivirals [65] and prophylactics.

## 6. *Epstein–Barr Virus*

*Epstein–Barr virus* (EBV) is a human herpesvirus that was discovered in continuously growing tumor cells derived from African patients with Burkitt′s lymphoma [66]. EBV infects the world′s population subclinically during childhood and thereafter remains in the body for life [67]. The virus targets B lymphocytes and epithelial cells [68,69] and possesses a unique position among human *Herpesviruses* for being capable of forming a latent infection whereby complete copies of the virus genome persist in growth transformed cells [66]. EBV can be spread primarily through saliva but also by blood or sexual intercourse [70,71]. EBV is a tumorigenic virus and is associated with a variety of diseases, including infectious mononucleosis, EBV-associated hemophagocytic syndrome, and gastric carcinoma [72,73]. Several animal species, including primates [74,75,76], severe combined immune-deficiency (SCID) mice [77], transgenic animals [78,79,80,81,82], and rabbits [83,84,85] have been used in EBV infection studies and these models of EBV infection have helped our understanding of the EBV infection process, pathogenic mechanism, and its prevention and treatment [27]. However, the biological characteristics of EBV, precisely its role in tumorigenesis, are yet to be defined due to the lack of an ideal animal model. Therefore, a suitable experimental EBV animal model is essential. A recent study demonstrated that tree shrews could be experimentally infected with EBV, which was confirmed by an increased EBV copy number, the detection of EBV-related gene expression, and the induction of extensive anti-EBV antibodies in EBV-infected tree shrews [27]. Pathological changes, including splenic corpuscle hyperplasia in the spleen and the infiltration of inflammatory cells into the liver and mesenteric lymph nodes, were also observed. In addition, in situ hybridization and immunohistochemical staining showed the presence of EBV-encoded nuclear RNA (EBER)-, latent membrane protein 1 (LMP1)-, and EBV-encoded nuclear antigen 2 (EBNA2)- positive cells in the spleens and mesenteric lymph nodes of some EBV-infected tree shrews [27]. Therefore, the tree shrew is a potentially suitable small animal that is susceptible to experimental EBV infection, opening the possibility for developing it as an alternative animal model for EBV infection study.

## 7. Conclusions

Recent work has demonstrated the potential value of the *Tupaia* as an animal model for many important human viral pathogens. No animal model is perfect; every animal model has its own merits and demerits, which is also true for the *Tupaia*. Currently, individual variability and the lack of analytical tools hamper the smooth application of the *Tupaia* as an animal model in viral infections. However, the *Tupaia* whole genome sequence data has now been released and the *Tupaia* database of both genomic DNA and mRNA, and total RNA sequencing has been constructed (by our group and others). Therefore, analytical tools such as antibodies, PCR, etc., could be developed from this sequence database, which will significantly improve the application of the *Tupaia* model in viral infection studies exploring pathogenesis and immune response. This will provide a better understanding of viral pathogenesis and host response, and thus facilitate the development of better therapeutic and prophylactic strategies. Additionally, *Tupaia* are small-sized, easy to breed and handle, and economical to house in animal facilities; the use of genetically inbred strains may also reduce individual variability. Thus, the *Tupaia* model could be a cornerstone of biomedical research and human viral infection studies in the near future.

## References

[1] Kawai T., Akira S. (2011). Toll-like receptors and their crosstalk with other innate receptors in infection and immunity. Immunity.

[2] Pandey S., Kawai T., Akira S. (2014). Microbial sensing by Toll-like receptors and intracellular nucleic acid sensors. Cold Spring Harb. Perspect. Biol..

[3] Akira S., Uematsu S., Takeuchi O. (2006). Pathogen recognition and innate immunity. Cell.

[4] Takeuchi O., Akira S. (2010). Pattern recognition receptors and inflammation. Cell.

[5] Sun L., Wu J., Du F., Chen X., Chen Z.J. (2013). Cyclic GMP-AMP synthase is a cytosolic DNA sensor that activates the type I interferon pathway. Science.

[6] Civril F., Deimling T., de Oliveira Mann C.C., Ablasser A., Moldt M., Witte G., Hornung V., Hopfner K.-P. (2013). Structural mechanism of cytosolic DNA sensing by cGAS. Nature.

[7] Tsukiyama-Kohara K., Kohara M. (2014). *Tupaia belangeri* as an experimental animal model for viral infection. Exp. Anim..

[8] Peng Y.Z., Ye Z.Z., Zou R.J., Wang Y.X., Tian B.P., Ma Y.Y., Shi L.M. (1991). Biology of Chinese Tree Shrews (Tupaia Belangeri Chinensis).

[9] Fan Y., Huang Z.Y., Cao C.C., Chen C.S., Chen Y.X., Fan D.D., He J., Hou H.L., Hu L., Hu X.T. (2013). Genome of the Chinese tree shrew. Nat. Commun..

[10] Sanada T., Tsukiyama-Kohara K., Shin-I T., Yamamoto N., Kayesh M.E.H., Yamane D., Takano J.-I., Shiogama Y., Yasutomi Y., Ikeo K. (2019). Construction of complete *Tupaia belangeri* transcriptome database by whole-genome and comprehensive RNA sequencing. Sci. Rep..

[11] Hai-Ying C., Nagano K., Ezzikouri S., Yamaguchi C., Kayesh M.E.H., Rebbani K., Kitab B., Nakano H., Kouji H., Kohara M. (2016). Establishment of an intermittent cold stress model using *Tupaia belangeri* and evaluation of compound C737 targeting neuron-restrictive silencer factor. Exp. Anim..

[12] Lee K.S., Huang X., Fitzpatrick D. (2016). Topology of ON and OFF inputs in visual cortex enables an invariant columnar architecture. Nature.

[13] Walter E., Keist R., Niederöst B., Pult I., Blum H.E. (1996). Hepatitis B virus infection of tupaia hepatocytes in vitro and in vivo. Hepatology.

[14] Yang C., Ruan P., Ou C., Su J., Cao J., Luo C., Tang Y., Wang Q., Qin H., Sun W. (2015). Chronic hepatitis B virus infection and occurrence of hepatocellular carcinoma in tree shrews (*Tupaia belangeri chinensis*). Virol. J..

[15] Sanada T., Tsukiyama-Kohara K., Yamamoto N., Ezzikouri S., Benjelloun S., Murakami S., Tanaka Y., Tateno C., Kohara M. (2016). Property of hepatitis B virus replication in *Tupaia belangeri* hepatocytes. Biochem. Biophys. Res. Commun..

[16] Kayesh M.E.H., Ezzikouri S., Chi H., Sanada T., Yamamoto N., Kitab B., Haraguchi T., Matsuyama R., Nkogue C.N., Hatai H. (2017). Interferon-β response is impaired by hepatitis B virus infection in *Tupaia belangeri*. Virus Res..

[17] Xu X., Chen H., Cao X., Ben K. (2007). Efficient infection of tree shrew (*Tupaia belangeri*) with hepatitis C virus grown in cell culture or from patient plasma. J. Gen. Virol..

[18] Amako Y., Tsukiyama-Kohara K., Katsume A., Hirata Y., Sekiguchi S., Tobita Y., Hayashi Y., Hishima T., Funata N., Yonekawa H. (2010). Pathogenesis of hepatitis C virus infection in *Tupaia belangeri*. J. Virol..

[19] Kayesh M.E.H., Ezzikouri S., Sanada T., Chi H., Hayashi Y., Rebbani K., Kitab B., Matsuu A., Miyoshi N., Hishima T. (2017). Oxidative stress and immune responses during hepatitis C virus infection in *Tupaia belangeri*. Sci. Rep..

[20] Yu W., Yang C., Bi Y., Long F., Li Y., Wang J., Huang F. (2016). Characterization of hepatitis E virus infection in tree shrew (*Tupaia belangeri chinensis*). BMC Infect. Dis..

[21] Yang Z.F., Zhao J., Zhu Y.T., Wang Y.T., Liu R., Zhao S.S., Li R.F., Yang C.G., Li J.Q., Zhong N.S. (2013). The tree shrew provides a useful alternative model for the study of influenza H1N1 virus. Virol. J..

[22] Li R., Yuan B., Xia X., Zhang S., Du Q., Yang C., Li N., Zhao J., Zhang Y., Zhang R. (2018). Tree shrew as a new animal model to study the pathogenesis of avian influenza (H9N2) virus infection. Emerg. Mmicrobes Infect..

[23] Sanada T., Yasui F., Honda T., Kayesh M.E.H., Takano J.I., Shiogama Y., Yasutomi Y., Tsukiyama-Kohara K., Kohara M. (2019). Avian H5N1 influenza virus infection causes severe pneumonia in the Northern tree shrew (*Tupaia belangeri*). Virology.

[24] Kayesh M.E.H., Kitab B., Sanada T., Hayasaka D., Morita K., Kohara M., Tsukiyama-Kohara K. (2017). Susceptibility and initial immune response of *Tupaia belangeri* cells to dengue virus infection. Infect. Genet. Evol..

[25] Zhang N.N., Zhang L., Deng Y.Q., Feng Y., Ma F., Wang Q., Ye Q., Han Y., Sun X., Zhang F.C. (2019). Zika virus infection in *Tupaia belangeri* causes dermatological manifestations and confers protection against secondary infection. J. Virol..

[26] Zhang L., Shen Z.L., Feng Y., Li D.Q., Zhang N.N., Deng Y.Q., Qi X.-P., Sun X.M., Dai J.-J., Yang C.-J. (2019). Infectivity of Zika virus on primary cells support tree shrew as animal model. Emerg. Microbes Infect..

[27] Wang Z., Yi X., Du L., Wang H., Tang J., Wang M., Qi C., Li H., Lai Y., Xia W. (2017). A study of Epstein-Barr virus infection in the Chinese tree shrew (*Tupaia belangeri chinensis*). Virol. J..

[28] Li L., Li Z., Wang E., Yang R., Xiao Y., Han H., Lang F., Li X., Xia Y., Gao F. (2016). Herpes simplex virus 1 infection of tree shrews differs from that of mice in the severity of acute infection and viral transcription in the peripheral nervous system. J. Virol..

[29] Li J.P., Liao Y., Zhang Y., Wang J.J., Wang L.C., Feng K., Li Q.H., Liu L.D. (2014). Experimental infection of tree shrews (*Tupaia belangeri*) with Coxsackie virus A16. Zool. Res..

[30] Xu L., Yu D., Fan Y., Peng L., Wu Y., Yao Y.G. (2016). Loss of RIG-I leads to a functional replacement with MDA5 in the Chinese tree shrew. Proc. Natl. Acad. Sci. USA.

[31] Bhatt S., Gething P.W., Brady O.J., Messina J.P., Farlow A.W., Moyes C.L., Drake J.M., Brownstein J.S., Hoen A.G., Sankoh O. (2013). The global distribution and burden of dengue. Nature.

[32] Simmons C.P., Farrar J.J., van Vinh Chau N., Wills B. (2012). Dengue. N. Engl. J. Med..

[33] Chan K.W.K., Watanabe S., Kavishna R., Alonso S., Vasudevan S.G. (2015). Animal models for studying dengue pathogenesis and therapy. Antiviral Res..

[34] Shi Y.J., Jiang Z.Y., Zeng K. (2006). Effect of IL-6 and TNF-alpha on dengue virus infection of human dendritic cells. Xi Bao Yu Fen Zi Mian Yi Xue Za Zhi.

[35] Tsai Y.T., Chang S.Y., Lee C.N., Kao C.L. (2009). Human TLR3 recognizes dengue virus and modulates viral replication in vitro. Cell. Microbiol..

[36] Liang Z., Wu S., Li Y., He L., Wu M., Jiang L., Feng L., Zhang P., Huang X. (2011). Activation of Toll-like receptor 3 impairs the dengue virus serotype 2 replication through induction of IFN-β in cultured hepatoma cells. PLoS ONE.

[37] Sun B., Sundström K.B., Chew J.J., Bist P., Gan E.S., Tan H.C., Goh K.C., Chawla T., Tang C.K., Ooi E.E. (2017). Dengue virus activates cGAS through the release of mitochondrial DNA. Sci. Rep..

[38] Hamel R., Dejarnac O., Wichit S., Ekchariyawat P., Neyret A., Luplertlop N., Perera-Lecoin M., Surasombatpattana P., Talignani L., Thomas F. (2015). Biology of Zika virus infection in human skin cells. J. Virol..

[39] Dick G.W.A., Kitchen S.F., Haddow A.J. (1952). Zika virus. I. Isolations and serological specificity. Trans. R. Soc. Trop. Med. Hyg..

[40] Bogoch I.I., Brady O.J., Kraemer M.U.G., German M., Creatore M.I., Kulkarni M.A., Brownstein J., Mekaru S.R., Hai S.I., Groot E. (2016). Anticipating the international spread of Zika virus from Brazil. Lancet.

[41] Imperato P.J. (2016). The convergence of a virus, mosquitoes, and human travel in globalizing the Zika epidemic. J. Community Health.

[42] Musso D., Gubler D.J. (2016). Zika Virus. Clin. Microbiol. Rev..

[43] Pierson T.C., Diamond M.S. (2018). The emergence of Zika virus and its new clinical syndromes. Nature.

[44] Morrison T.E., Diamond M.S. (2017). Animal models of Zika virus infection, pathogenesis, and immunity. J. Virol..

[45] Cerbino-Neto J., Mesquita E.C., Souza T.M., Parreira V., Wittlin B.B., Durovni B., Lemos M.C., Vizzoni A., Bispo de Filippis A.M., Sampaio S.A. (2016). Clinical manifestations of Zika virus infection, Rio de Janeiro, Brazil, 2015. Emerg. Infect. Dis..

[46] Lavanchy D. (2009). The global burden of hepatitis C. Liver Int..

[47] Mohd Hanafiah K., Groeger J., Flaxman A.D., Wiersma S.T. (2013). Global epidemiology of hepatitis C virus infection: New estimates of age-specific antibody to HCV seroprevalence. Hepatology.

[48] Mercer D.F., Schiller D.E., Elliott J.F., Douglas D.N., Hao C., Rinfret A., Addison W.R., Fischer K.P., Churchill T.A., Lakey J.R. (2001). Hepatitis C virus replication in mice with chimeric human livers. Nat. Med..

[49] Dorner M., Horwitz J.A., Robbins J.B., Barry W.T., Feng Q., Mu K., Jones C.T., Schoggins J.W., Catanese M.T., Burton D.R. (2011). A genetically humanized mouse model for hepatitis C virus infection. Nature.

[50] Takano T., Tsukiyama-Kohara K., Hayashi M., Hirata Y., Satoh M., Tokunaga Y., Tateno C., Hayashi Y., Hishima T., Funata N. (2011). Augmentation of DHCR24 expression by hepatitis C virus infection facilitates viral replication in hepatocytes. J. Hepatol..

[51] De Maria N., Colantoni A., Fagiuoli S., Liu G.J., Rogers B.K., Farinati F., Van Thiel D.H., Floyd R.A. (1996). Association between reactive oxygen species and disease activity in chronic hepatitis C. Free Radic. Biol. Med..

[52] Valgimigli L., Valgimigli M., Gaiani S., Pedulli G.F., Bolondi L. (2000). Measurement of oxidative stress in human liver by EPR spin-probe technique. Free Radic. Res..

[53] Valgimigli M., Valgimigli L., Trerè D., Gaiani S., Pedulli G.F., Gramantieri L., Bolondi L. (2002). Oxidative stress EPR measurement in human liver by radical-probe technique. Correlation with etiology, histology and cell proliferation. Free Radic. Res..

[54] Tsukiyama-Kohara K. (2012). Role of oxidative stress in hepatocarcinogenesis induced by hepatitis C virus. Int. J. Mol. Sci..

[55] Saito M., Kohara M., Tsukiyama-Kohara K. (2012). Hepatitis C virus promotes expression of the 3beta-hydroxysterol delta24-reductase through Sp1. J. Med. Virol..

[56] Ezzikouri S., Kimura K., Sunagozaka H., Kaneko S., Inoue K., Nishimura T., Hishima T., Kohara M., Tsukiyama-Kohara K. (2015). Serum DHCR24 auto-antibody as a new biomarker for progression of hepatitis C. EBioMedicine.

[57] Ding Q., Cao X., Lu J., Huang B., Liu Y.J., Kato N., Shu H.B., Zhong J. (2013). Hepatitis C virus NS4B blocks the interaction of STING and TBK1 to evade host innate immunity. J. Hepatol..

[58] Lee W.M. (1997). Hepatitis B virus infection. N. Engl. J. Med..

[59] Ganem D., Prince A.M. (2004). Hepatitis B virus infection--natural history and clinical consequences. N. Engl. J. Med..

[60] Schweitzer A., Horn J., Mikolajczyk R.T., Krause G., Ott J.J. (2015). Estimations of worldwide prevalence of chronic hepatitis B virus infection: A systematic review of data published between 1965 and 2013. Lancet.

[61] Ruan P., Yang C., Su J., Cao J., Ou C., Luo C., Tang Y., Wang Q., Yang F., Shi J. (2013). Histopathological changes in the liver of tree shrew (*Tupaia belangeri chinensis*) persistently infected with hepatitis B virus. Virol. J..

[62] Kouwaki M., Fukushima Y., Daito T., Sanada T., Yamamoto N., Mifsud E.J., Leong C.R., Tsukiyama-Kohara K., Kohara M., Matsumoto M. (2016). Extracellular vesicles including exosomes regulate innate immune responses to hepatitis B virus infection. Front. Immunol..

[63] Ma Z., Cao Q., Xiong Y., Zhang E., Lu M. (2018). Interaction between hepatitis B virus and Toll-like receptors: Current status and potential therapeutic use for chronic hepatitis B. Vaccines.

[64] Huang Y.W., Hsu C.K., Lin S.C., Wei S.C., Hu J.T., Chang H.Y., Liang C.W., Chen D.S., Chen P.J., Hsu P.N. (2014). Reduced toll-like receptor 9 expression on peripheral CD14+ monocytes of chronic hepatitis B patients and its restoration by effective therapy. Antivir. Ther..

[65] Sanada T., Yamamoto N., Kayesh M.E.H., Tsukiyama-Kohara K., Hasegawa H., Miyazaki T., Takano J.-I., Shiogama Y., Yasutomi Y., Goh Y. (2019). Intranasal vaccination with HBs and HBc protein combined with carboxyl vinylpolymer induces strong neutralizing antibody, anti-HBs IgA, and IFNG response. Biochem. Biophys. Res. Commun..

[66] Ooka T. (1985). The molecular biology of Epstein-Barr virus. Biomed. Pharmacother..

[67] Crawford D.H. (2001). Biology and disease associations of Epstein-Barr virus. Philos. Trans. R. Soc. Lond. B Biol. Sci..

[68] Mrozek-Gorska P., Buschle A., Pich D., Schwarzmayr T., Fechtner R., Scialdone A., Hammerschmidt W. (2019). Epstein-Barr virus reprograms human B lymphocytes immediately in the prelatent phase of infection. Proc. Natl. Acad. Sci. USA.

[69] Chen J., Longnecker R. Epithelial cell infection by Epstein-Barr virus. FEMS Microbiol. Rev..

[70] Näher H., Gissmann L., Freese U.K., Petzoldt D., Helfrich S. (1992). Subclinical Epstein-Barr virus infection of both the male and female genital tract--indication for sexual transmission. J. Invest. Dermatol..

[71] Balfour H.H., Odumade O.A., Schmeling D.O., Mullan B.D., Ed J.A., Knight J.A., Vezina H.E., Thomas W., Hogquist K.A. (2013). Behavioral, virologic, and immunologic factors associated with acquisition and severity of primary Epstein-Barr virus infection in university students. J. Infect. Dis..

[72] Okano M. (2000). Haematological associations of Epstein-Bar virus infection. Baillieres Best Pract. Res. Clin. Haematol..

[73] Xu F.H., Xiong D., Xu Y.F., Cao S.M., Xue W.Q., Qin H.D., Liu W.S., Cao J.Y., Zhang Y., Feng Q.S. (2012). An epidemiological and molecular study of the relationship between smoking, risk of nasopharyngeal carcinoma, and Epstein-Barr virus activation. J. Natl. Cancer Inst..

[74] Shope T., Dechairo D., Miller G. (1973). Malignant lymphoma in cottontop marmosets after inoculation with Epstein-Barr virus. Proc. Natl. Acad. Sci. USA.

[75] Niedobitek G., Agathanggelou A., Finerty S., Tierney R., Watkins P., Jones E.L., Morgan A., Young L.S., Rooney N. (1994). Latent Epstein-Barr virus infection in cottontop tamarins. A possible model for Epstein-Barr virus infection in humans. Am. J. Pathol..

[76] Cleary M.L., Epstein M.A., Finerty S., Dorfman R.F., Bornkamm G.W., Kirkwood J.K., Morgan A.J., Sklar J. (1985). Individual tumors of multifocal EB virus-induced malignant lymphomas in tamarins arise from different B-cell clones. Science.

[77] Mosier D.E., Gulizia R.J., Baird S.M., Wilson D.B. (1988). Transfer of a functional human immune system to mice with severe combined immunodeficiency. Nature.

[78] Chijioke O., Muller A., Feederle R., Barros M.H., Krieg C., Emmel V., Marcenaro E., Leung C.S., Antsiferova O., Landtwing V. (2013). Human natural killer cells prevent infectious mononucleosis features by targeting lytic Epstein-Barr virus infection. Cell Rep..

[79] Wilson J.B., Bell J.L., Levine A.J. (1996). Expression of Epstein-Barr virus nuclear antigen-1 induces B cell neoplasia in transgenic mice. EMBO J..

[80] Hannigan A., Qureshi A.M., Nixon C., Tsimbouri P.M., Jones S., Philbey A.W., Wilson J.B. (2011). Lymphocyte deficiency limits Epstein-Barr virus latent membrane protein 1 induced chronic inflammation and carcinogenic pathology in vivo. Mol. Cancer.

[81] Hartlage A.S., Liu T., Patton J.T., Garman S.L., Zhang X., Kurt H., Lozanski G., Lustberg M.E., Caligiuri M.A., Baiocchi R.A. (2015). The Epstein-Barr Virus Lytic Protein BZLF1 as a Candidate Target Antigen for Vaccine Development. Cancer Immunol. Res..

[82] Kulwichit W., Edwards R.H., Davenport E.M., Baskar J.F., Godfrey V., Raab-Traub N. (1998). Expression of the Epstein-Barr virus latent membrane protein 1 induces B cell lymphoma in transgenic mice. Proc. Natl. Acad. Sci. USA.

[83] Takashima K., Ohashi M., Kitamura Y., Ando K., Nagashima K., Sugihara H., Okuno K., Sairenji T., Hayashi K. (2008). A new animal model for primary and persistent Epstein-Barr virus infection: Human EBV-infected rabbit characteristics determined using sequential imaging and pathological analysis. J. Med. Virol..

[84] Okuno K., Takashima K., Kanai K., Ohashi M., Hyuga R., Sugihara H., Kuwamoto S., Kato M., Sano H., Sairenji T. (2010). Epstein-Barr virus can infect rabbits by the intranasal or peroral route: An animal model for natural primary EBV infection in humans. J. Med. Virol..

[85] Khan G., Ahmed W., Philip P.S., Ali M.H., Adem A. (2015). Healthy rabbits are susceptible to Epstein-Barr virus infection and infected cells proliferate in immunosuppressed animals. Virol. J..

